# A high-precision image-guided platform for studying spinal cord toxicity under ultra-high dose rate electron irradiation

**DOI:** 10.1088/1361-6560/ae56cb

**Published:** 2026-04-08

**Authors:** Banghao Zhou, Lixiang Guo, Yi-Chun Tsai, Albert van der Kogel, John W Wong, Iulian Iordachita, Kai Jiang, Weiguo Lu, Paul M Medin, Ken Kang-Hsin Wang

**Affiliations:** 1 Biomedical Imaging and Radiation Technology Laboratory (BIRTLab), Department of Radiation Oncology, University of Texas Southwestern Medical Center, Dallas, TX, United States of America; 2 Department of Human Oncology, School of Medicine and Public Health, University of Wisconsin, Madison, WI, United States of America; 3 Department of Radiation Oncology and Molecular Radiation Sciences, School of Medicine, Johns Hopkins University, Baltimore, MD, United States of America; 4 Department of Mechanical Engineering, Whiting School of Engineering, Johns Hopkins University, Baltimore, MD, United States of America; 5 Department of Radiation Oncology, University of Texas Southwestern Medical Center, Dallas, TX, United States of America

**Keywords:** FLASH radiotherapy, preclinical radiation research, late toxicity, spinal cord

## Abstract

**Objective.:**

While FLASH radiotherapy (FLASH-RT) is recognized for normal tissue sparing, its effect in mitigating the toxicity of late-responding organs remains uncertain, limiting clinical adoption. With its clinical importance and steep dose-response, spinal cord is an ideal model for evaluating the FLASH effect on late toxicity. This work introduces a robust image-guided research platform for high-precision irradiation at both conventional (CONV) and ultra-high dose rates (UHDR) to enable FLASH late toxicity studies using a rat spinal cord model.

**Approach.:**

A modified LINAC was employed to irradiate the C1–T2 rat spinal cord with 18 MeV UHDR and CONV beams. A custom rat immobilization device, a portable x-ray imaging system, and an ionchamber-based UHDR output monitoring system were integrated to ensure accurate C1–T2 localization and precise dose delivery. A Monte Carlo (MC) dose engine was developed to provide accurate dosimetry and support the interpretation of *in vivo* results. Scintillator measurements at UHDR were performed within the spinal cord to verify MC results and the precision of our platform.

**Results.:**

We observed submillimeter deviation in C1–T2 localization between 2D x-ray and 3D cone beam computed tomography imaging, as well as between pre- and post-irradiation 2D x-ray assessments. Ion chamber readings showed linear correlation with UHDR output (*R*^2^ = 1). MC calculations indicated uniform irradiation (<5% non-uniformity) along the central ~13 mm cord, avoiding dose-volume effects. Our CONV beam exhibited dose distribution close to that of the UHDR beam, with differences < 3%, isolating dose rate as the only variable. Scintillator-measured dose agreed with MC within 4%, with a 100% gamma passing rate (2%/2 mm), confirming both MC accuracy and the platform’s high-precision delivery.

**Significance.:**

We developed the first comprehensive, image-guided preclinical platform for accurate UHDR and CONV irradiation to investigate FLASH-mitigated spinal cord toxicity in rats. This work thus establishes a robust foundation for systematic evaluation of the FLASH effect in late-responding organs and for assessing relevant clinical applicability of FLASH-RT.

## 1. Introduction

FLASH radiotherapy (FLASH-RT), characterized by ultra-high dose rates (UHDR, >40 Gy s^−1^), offers transformative potential to reduce normal tissue toxicity without compromising tumor control, known as the FLASH effect (Favaudon *et al* 2014). Despite promising preclinical evidence, key barriers hinder clinical translation of FLASH-RT. Most studies focus on acute toxicity, while its effects on sparing late-responding tissues remain largely unexamined (Hageman *et al* 2022, Taylor *et al* 2022, Vozenin *et al* 2022).

The spinal cord, frequently constituting the most critical late-responding organ, has a well described steep dose-response curve. Radiation exceeding its tolerance significantly increases the risk of myelopathy, leading to paresis, which can be easily discerned (Schultheiss and Stevens 2022). Its dose-limiting nature and clinical relevance make the spinal cord an ideal model for investigating whether the FLASH effect is sustained in late-responding organs (Ursino *et al* 2023).

Numerous studies have characterized spinal cord responses to conventional dose rate (CONV-RT) across various animal models, with the vast majority performed in rats. Studies using rats have generated a large body of clinically relevant data on the influence of dose, fractionation, re-irradiation, and volume on the tolerance of the spinal cord (Schultheiss and Stevens 2022). However, comparable data under FLASH-RT conditions remain unavailable, and no dedicated platform has been established to enable precise spinal cord irradiation in rats at both UHDR and CONV dose rates.

To address this critical unmet need, we propose to use rats as a clinically relevant model to investigate whether FLASH-RT can mitigate radiation-induced spinal cord injury. To ensure the robustness and reproducibility of the proposed study, it is essential to employ a high-precision research platform capable of accurately delivering both UHDR and CONV irradiation, under comparable beam characteristics to the rat spinal cord, while also enabling precise *in vivo* dosimetry. Previous works by Bijl *et al* (2002) showed that under CONV irradiation, spinal cord tolerance exhibits a strong dose-volume effect, with the median effective dose (ED_50_, the dose causing paresis in 50% of animals) increasing markedly when the irradiated length was <8 mm. To avoid this known confounding factor, many studies therefore have adopted the cervicothoracic (C1–T2) region (>20 mm in length) for spinal cord toxicity studies. For our proposed study, we follow this established experimental design strategy to ensure that potential FLASH-related sparing effects are evaluated under conditions where the known influence of volumedependent tolerance is eliminated. To this end, we have developed a LINAC-based platform that utilizes 18 MeV electrons to uniformly irradiate the C1–T2 region of the rat spinal cord in both UHDR and CONV modes.

Given the rapid delivery of the UHDR beam (~2 Gy per pulse at 180 Hz) and steep dose-response curve of the spinal cord (Bijl *et al* 2002), achieving precise dose delivery and dosimetry is essential to determine reproducible dose-response relationships. Specifically, an external pulse control system was implemented to control LINAC pulse delivery at UHDR. Moreover, an ionization chamber (IC) positioned beneath the electron applicator was utilized to monitor Bremsstrahlung and scattered radiation in real-time, serving as a surrogate for UHDR output monitoring. To achieve precise animal setup and spinal cord localization for irradiation, we designed a custom rat immobilization device integrated with a portable x-ray imaging system, allowing accurate and reproducible localization of the spine under image guidance.

We aim to evaluate the extent of normal tissue sparing of FLASH-RT by conducting studies in which dose rate is the only variable. Achieving this requires similar beam characteristics between the UHDR and CONV beams, as well as an accurate dose engine to generate dosimetric plans for evaluating both 18 MeV UHDR and CONV spinal cord irradiation. We developed a Geant4-GAMOS Monte Carlo (MC) dose engine that precisely models the electron beam. Our dose engine was also employed to quantify electron dose inhomogeneities and guide the experimental design to avoid the dose-volume effects, which could confound the assessment of the FLASH effect in sparing spinal cord toxicity. We further validated the MC-calculated FLASH dose and dose rates using image-guided scintillator measurements sampled at 1000 Hz within the spinal cord.

This work presents the first image-guided FLASH platform enabling precise rat spinal cord irradiation at both UHDR and CONV dose rates, providing a valuable tool to evaluate FLASH-RT’s potential to reduce late toxicity and support safe clinical translation.

## 2. Methods and materials

### 2.1. Irradiation setup

A Varian 21EX LINAC (Varian Medical Systems, Palo Alto, CA, USA) was modified to deliver 18 MeV electron beams for rat spinal cord irradiation at both UHDR and CONV dose rates. A posterior-anterior (PA) shoot-through geometry, a 2 × 1 cm^2^ field size, and a 100 cm source-to-surface distance (SSD) were employed to enable uniform coverage of the C1–T2 spinal cord segment. UHDR delivery was controlled using an external pulse control system, enabling modulation of pulse number (Zhou *et al* 2025). For CONV mode, standard monitor unit control was used. Female Sprague Dawley rats (aged 9–10 weeks, Charles River Laboratories, Wilmington, MA, USA) under isoflurane anesthesia were used for the irradiation studies in accordance with the Institutional Animal Care and Use Committee at The University of Texas Southwestern Medical Center (animal protocol number 2023–103504).

### 2.2. Image-guided animal positioning and target localization

#### 2.2.1. Image-guided system and setup procedure

**Figure 1.**
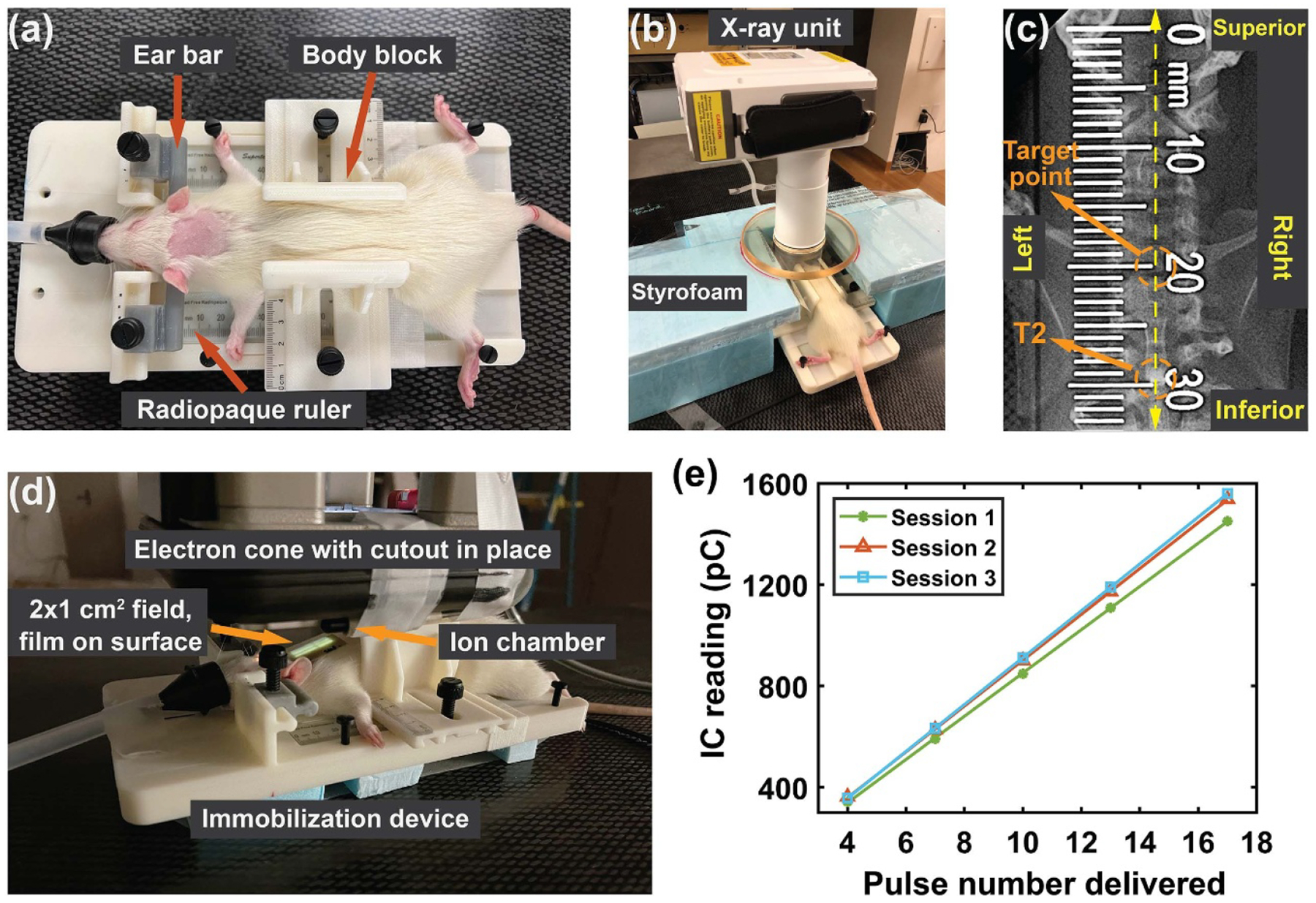
(a) Custom rat immobilization device. (b) Setup for x-ray imaging. (c) A representative x-ray image showing identification of the T2 vertebra and corresponding target point. (d) A rat positioned in the immobilization device for UHDR irradiation. The electron cone features a 2 × 1 cm^2^ cutout for C1–T2 irradiation. For each FLASH group, 3 rats were randomly selected to perform a sanity check of the surface dose rate using the film. A CC13 ion chamber was attached to the cone to measure the Bremsstrahlung and scattered radiation, enabling real-time UHDR output monitoring. (e) Ion chamber readings linearly correlated with the number of delivered pulses (*R*^2^ = 1) across 3 independent measurement sessions conducted over 2 weeks. The within-session standard deviation is smaller than symbol used in the figure. The observed session-to-session variation in slope reflects day-to-day fluctuations in absolute UHDR dose per pulse, which were confirmed by daily film dosimetry.

To ensure accurate positioning and target localization required for precise irradiation, image-guidance is necessary. Cone beam computed tomography (CBCT) is widely used as a standard on-board image-guidance modality in both clinical and preclinical RT. However, the LINAC used in this study does not have an integrated CBCT system. Moreover, even for LINACs equipped with CBCT, such systems are optimized for human-scale anatomy and are not well suited for routine small-animal imaging due to suboptimal resolution and imaging quality (Wong *et al* 2008, Brown *et al* 2022). Therefore, a custom 3D-printed rat immobilization device (figure 1(a)), along with a portable x-ray system (figure 1(b); DX3000, 65 kV, 2 mA, Dexcowin, Seoul, Republic of Korea), was developed to enable rapid image-guided animal positioning and target localization with high reproducibility and accuracy.

The immobilization device incorporated stereotaxic ear bars and lateral body supports to stabilize the head, neck, and torso. It also featured three parallel embedded radiopaque rulers to facilitate vertebral localization: one central ruler positioned beneath the animal for imaging, and two lateral rulers used for aligning the device with the LINAC light field crosshairs.

For target localization, the rat to be irradiated was first positioned on the immobilization device placed on the treatment couch (figure 1(a)), with the LINAC gantry angle fixed at 0° and the electron cone removed. An initial PA x-ray image was acquired using the portable x-ray unit with an array detector (Woodpecker Medical Instrument, Guilin, China) positioned beneath the device (figure 1(b)). Based on the image, the straightened C1–T2 spine was aligned with the distal ends of the horizontal marker lines on the central ruler, corresponding to the L–R midline of the immobilization device (S–I oriented yellow dashed line in figure 1(c)). When necessary, minor positional adjustments were performed, followed by confirmatory imaging.

Along the superior–inferior (S–I) direction, the T2 vertebra was first identified on the central ruler (e.g. at 30 mm in figure 1(c)) as it can be easily discerned on the x-ray image. The irradiation target point, defined as the transverse center of the spinal cord located 10 mm cranial to T2, was then identified on the same ruler (e.g. at 20 mm in figure 1(c)). The same longitudinal position is simultaneously marked on the two lateral rulers (figure 1(a)), enabling the imaging-defined target location on the central ruler to be transferred to a visually accessible reference for subsequent optical alignment with the LINAC crosshair. This target point lies near the midpoint of the ~20 mm long C1–T2 spinal segment and was designed to be aligned with the beam’s central axis (CAX) for irradiation, ensuring full coverage of the C1–T2 segment within the 2 × 1 cm^2^ field.

After confirming the target point location relative to the immobilization device using the central radiopaque ruler and 2D imaging, the x-ray unit and detector were removed. The couch was then translated to align the target point with the beam’s CAX, while the immobilization device remained fixed on the couch and only minor angular adjustments were applied when needed to fine-tune the alignment. This alignment was achieved and verified optically using the LINAC light field, such that in the L–R direction, the crossline crosshair coincided with the target point position marked on the lateral rulers, and in the S–I direction, the inline crosshair coincided with the device midline marker, which was temporarily exposed by removing the anesthesia hose and mask (figure 1(a)). Finally, the couch height was adjusted to achieve an SSD of 100 cm to the dorsal surface of the rat, with dorsal hair shaved during anesthesia induction. The electron cone was put back in position (figure 1(d)), and the setup was ready for irradiation.

#### 2.2.2. Validation for 2D x-ray image guidance and immobilization

In preclinical irradiation research, CBCT has been used for image guidance and is implemented on multiple small-animal irradiators (Wong *et al* 2008, Clarkson *et al* 2011, Brown *et al* 2022). To assess the agreement between our developed 2D x-ray image-guided target localization method and CBCT, CBCT imaging was performed on a nearby small-animal irradiator (X-RAD 225, Precision x-ray, Madison, CT, USA) and used as a validation reference.

Eight non-irradiated rats were used as an imaging validation cohort and underwent the 2D x-ray guided setup process described above in the LINAC vault. They were then transported together with the immobilization device to a nearby room with X-RAD 225 for CBCT scanning. Because the spine was fully straightened and aligned with the midline of the immobilization device, and the target point could be readily determined relative to the T2 vertebra, the T2 position was used as a representative landmark to evaluate C1–T2 alignment. The T2 positions identified on the CBCT images were compared with those determined on the 2D x-ray images to assess the agreement between our proposed 2D x-ray localization method and the commonly accepted CBCT approach (supplementary figure S1).

To further assess potential intra-fraction motion of the spinal cord, 12 irradiated rats underwent an additional post-irradiation x-ray imaging and for each rat, the T2 vertebra locations identified on the pre- and post-irradiation images were compared to evaluate any positional changes.

### 2.3. UHDR output monitoring

As the LINAC’s dose servo system was disabled to enable UHDR delivery (Rahman *et al* 2021, Zhou *et al* 2025), the inherent beam output regulation was no longer available (Greene and Williams 2017). To ensure accurate dose delivery, UHDR output was externally monitored daily and during irradiation for each rat to account for any output fluctuations.

This was accomplished by a two-step approach. First, the daily absolute dose per pulse (DPP), a direct measure of UHDR output, was determined using Gafchromic EBT-XD films (Ashland, Bridgewater, NJ, USA) irradiated under a reference condition (1 cm depth in solid water, 2 × 1 cm^2^ field size, 100 cm SSD) to account for day-to-day UHDR output variations. Next, an IC (CC13, IBA, Louvain-La-Neuve, Belgium) was mounted on the distal end of the electron cone and positioned beneath one lateral edge of the aperture (supplementary figure S2) to measure bremsstrahlung and scattered radiation, serving as a surrogate for monitoring relative changes in output during UHDR delivery. The daily output measurement by film, together with relative output monitoring by IC, was used to determine the absolute dose, which was then used as input to the MC dose calculations to obtain the *in vivo* dose for each irradiated rat.

The detailed procedure is provided as follows. Before each day’s animal irradiation session, 3 film measurements were acquired under the reference condition to establish the baseline DPP. Corresponding IC readings were recorded simultaneously to determine the baseline IC reading per pulse. During animal irradiation, the measured IC reading was compared to the baseline value to determine relative output variation. Using the baseline DPP together with the output variation, the actual DPP, or UHDR output, delivered to each irradiated animal was determined and subsequently used in the MC engine to calculate the dose delivered to each animal. All IC readings were corrected for temperature and pressure. The difference in IC readings between the solid water and rat irradiation setups was found negligible.

Both the film dosimetry and IC-based output monitoring methods were independently validated. EBT-XD films were selected for UHDR measurement due to their demonstrated superiority in dose rate independence (Romano *et al* 2022, Villoing *et al* 2022). Films were scanned 24 h post-irradiation using a photo flatbed scanner (Epson Expression 12000XL, Suwa, Nagano, Japan), and analyzed using in-house software with a dual-channel method (green and blue, Micke *et al* 2011). Film-measured doses were validated against the known doses delivered by the 18 MeV CONV beam in solid water, showing <3% deviations over the 0–40 Gy dose range. The IC method was validated across 3 independent measurement sessions over a 2-week period.

### 2.4. *In vivo* dose and dose rate determination

#### 2.4.1. MC engine

An MC engine was developed to compute *in vivo* dose and dose rate distributions (Zhou *et al* 2025). Using the Geant4-based GAMOS MC package (Arce *et al* 2014), the LINAC head geometry was explicitly simulated, and beam models were commissioned for both UHDR and CONV modes. Beam parameters including mean energy, energy spread, source emittance cone angle, and spot size were optimized to achieve <2% average absolute difference between MC-simulated and measured percent depth dose (PDD) and profiles at depths of 0–4 cm at 100 cm SSD for various field sizes. Phase-space files generated at 96 cm SSD below the electron applicator, along with animal CBCT scans, were used for subsequent *in vivo* dose and dose rate calculations.

A segmentation-based discrete material assignment strategy was employed to enable MC dose calculation based on CBCT images. Animal CBCT images were imported into the Eclipse (Varian, Palo Alto, CA, USA) for anatomical segmentation and then exported to GAMOS. Segmented regions included the body, spinal cord, esophagus, lung, and bone. In GAMOS, image voxels were assigned with Geant4 predefined materials according to their segmented tissue class, including G4_AIR for the esophagus, G4_BONE_CORTICAL_ICRP for bones, and G4_TISSUE_SOFT_ICRP for the spinal cord and soft tissues. The absolute doses in the MC calculation were obtained from raw values by applying a scaling factor, which was determined under the aforementioned reference condition as the ratio of the measured dose to the MC raw value. The statistical uncertainty of MC simulations was maintained below 3%.

#### 2.4.2. In vivo *dose distribution calculations*

To streamline the workflow of our spinal cord study while maintaining accuracy, we evaluated whether an averaged relative dose distribution derived from a representative cohort of non-irradiated rats of the same age and similar weight as the experimental groups could be applied to each irradiated rat. This approach avoids the need for CBCT-based MC calculations for every individual animal. The non-irradiated rats of the imaging validation cohort were utilized for this purpose. Each rat had undergone the standard setup applied to irradiated rats as described in section 2.2.

For each rat, *in vivo* dose distributions were calculated for both UHDR and CONV scenarios and normalized to 100% at the target point. The longitudinal profile along the C1–T2 spinal cord, the lateral profile across the target point in the L–R direction, and the PDD_TP_ (PDD along the CAX normalized to the spinal cord target point) were extracted. We then evaluated whether the averaged dose distribution from this cohort could be used for subsequent irradiated rats by assessing inter-animal anatomical variability in these profiles and PDD_TP_, with dosimetric variations quantified by their standard deviations.

### 2.5. Image-guided scintillator dosimetry

A HYPERSCINT RP-FLASH scintillation dosimetry system (Medscint Inc., Quebec, Canada) was used to validate *in vivo* MC dose/dose rate calculations. The detector probe features a cylindrical sensitive volume of 1 mm diameter and 3 mm length and operates at a 1000 Hz sampling frequency, ensuring sufficient spatial and inter-pulse resolution for UHDR measurements. Prior to use, the scintillator system was calibrated under UHDR irradiation, with a dose error of <3% compared to film measurements up to 40 Gy. The impact of the differences in beam energy and field size between the calibration and this measurement on dose accuracy was within 2% (Guo *et al* 2025). As shown in supplementary figure S3, the raw signal acquired by the scintillator at a 1000 Hz clearly resolves individual pulses of the 18 MeV UHDR beam delivered at a pulse repetition frequency (PRF) of 180 Hz, from which DPP can be extracted as previously reported by Guo *et al* (2025).

A rat was euthanized and underwent the standard irradiation setup and CBCT imaging within 2 h postmortem, during which the carcass became rigid while tissue integrity was preserved. The carcass was transected at the L3 vertebral level to create an access opening to the spinal canal. This opening was located more than 2 cm away from the irradiation field and therefore did not affect the dose distribution. The plastic scintillator probe, enclosed in a 6 Fr catheter, was inserted cranially along the spinal canal and positioned within the C1–T2 region.

The detector was advanced cranially along the natural curvature of the spinal canal under minimal mechanical resistance, without forceful dilation of the canal. Fine positioning was achieved using a linear translation stage (10 *μ*m resolution, XR25P, Thorlabs, Newton, NJ), and detector locations were verified by the portable x-ray imaging system. Measurements of dose and dose rates under UHDR irradiation were acquired at multiple longitudinal positions and compared with MC calculations for validation. Because both the plastic scintillator and the polyurethane catheter are near tissue-equivalent, the impact of tissue displacement on dose distribution is minimal and not explicitly corrected.

### 2.6. Overall experimental workflow

**Figure 2.**
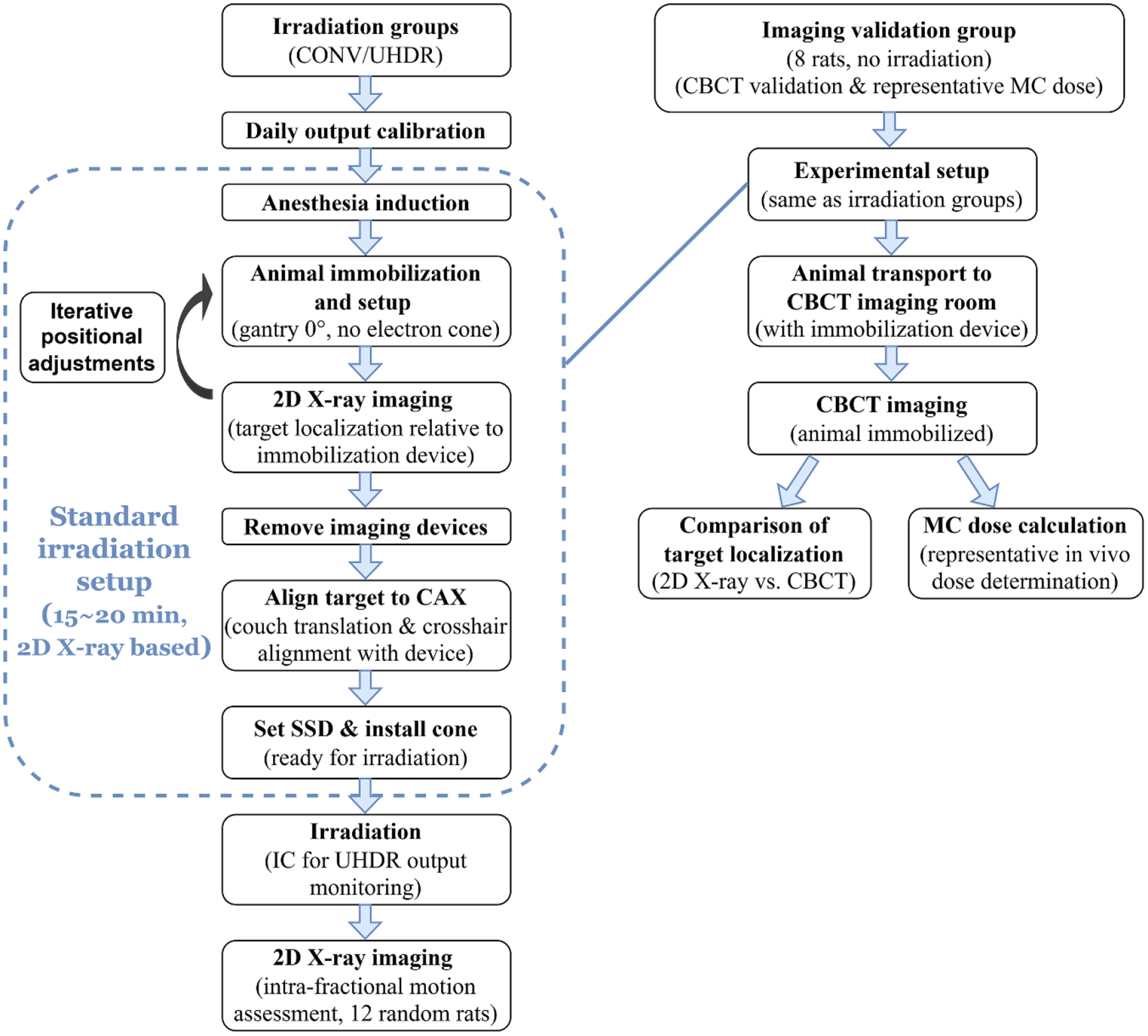
Overall experimental workflow for irradiation and imaging validation groups.

Figure 2 illustrates the overall experimental workflow for both the irradiation and imaging validation groups, as described in sections 2.1–2.5.

## 3. Results

### 3.1. Assessment of the setup agreement and intra-fractional motion

For the 8 non-irradiated rats used for setup evaluation (figure 2), the T2 locations identified using 2D x-ray imaging showed good agreement with those determined from CBCT (table 1). The positional differences are within sub-millimeter, with mean offsets of 0.3 ± 0.3 mm (S–I) and 0.2 ± 0.1 mm (L–R), even when accounting for potential offsets introduced during animal transport between the 2D x-ray and CBCT imaging systems. Comparisons of pre- and post-irradiation x-ray images in the 12 irradiated rats showed minimal intra-fractional T2 displacements of 0.3 ± 0.6 mm and 0.2 ± 0.1 mm in S–I and L–R directions, respectively. These results confirm the accurate 2D image-guided setup and effective immobilization, providing the foundation for high-precision irradiation delivery.

### 3.2. IC-based UHDR output monitoring

Across the 3 independent measurement sessions over a 2 week period, IC readings exhibited strong linearity with the number of delivered pulses within each session (*R*^2^ = 1, figure 1(e)). A difference in the absolute slope between sessions was observed, indicating day-to-day variation in the absolute DPP. Corresponding film measurements further confirmed the linear correlation between IC readings and delivered dose (data not shown), supporting the reliability of the IC method for relative output monitoring during experiments.

### 3.3. MC *in vivo* dose calculation

#### 3.3.1. Beam data and MC model

**Figure 3.**
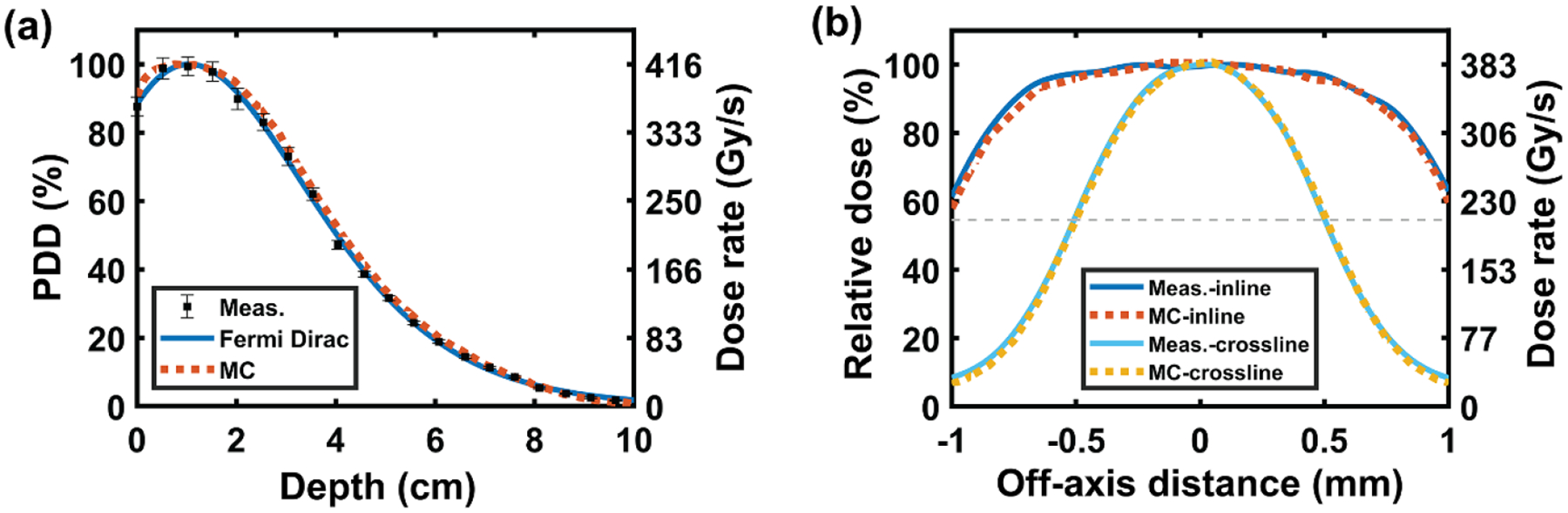
Comparison between MC-simulated and film-measured beam data for the 18 MeV UHDR beam in solid water using a 2 × 1 cm^2^ field at 100 cm SSD. (a) PDD and depth dose rates along the beam CAX. The film-measured PDD data were fitted with a Fermi–Dirac distribution multiplied by a 3rd-degree polynomial with *R*^2^ ⩾ 0.99 to generate continuous PDD curves. (b) Relative dose and dose rate profiles at 2 cm depth in both crossline and inline directions. The average absolute differences between MC simulations and measurements (Meas.) were within 2%.

Along the UHDR beam’s CAX, the PDD at 2 × 1 cm^2^ remained > 90% and the dose rate >383 Gy s^−1^ within the first 2 cm depth in solid water (figure 3(a)). At 2 cm depth, the dose rate profile sustained >209 Gy s^−1^ across the 2 × 1 cm^2^ field (the gray line in figure 3(b)). These beam characteristics confirm that the 18 MeV UHDR beam provides sufficient FLASH dose rates for C1–T2 spinal cord irradiation.

For the UHDR spinal cord irradiation using a 2 × 1 cm^2^ field, the average absolute differences between MC calculation and film measurements were 1.6% for the PDD (figure 3(a)), 1.0% for the crossline profile, and 1.4% for the inline profile (figure 3(b)). These results confirm the accuracy of the MC beam model and support its use for *in vivo* spinal cord dosimetry.

#### 3.3.2. Dose distribution of C1–T2 UHDR irradiation and inter-animal deviation

**Figure 4.**
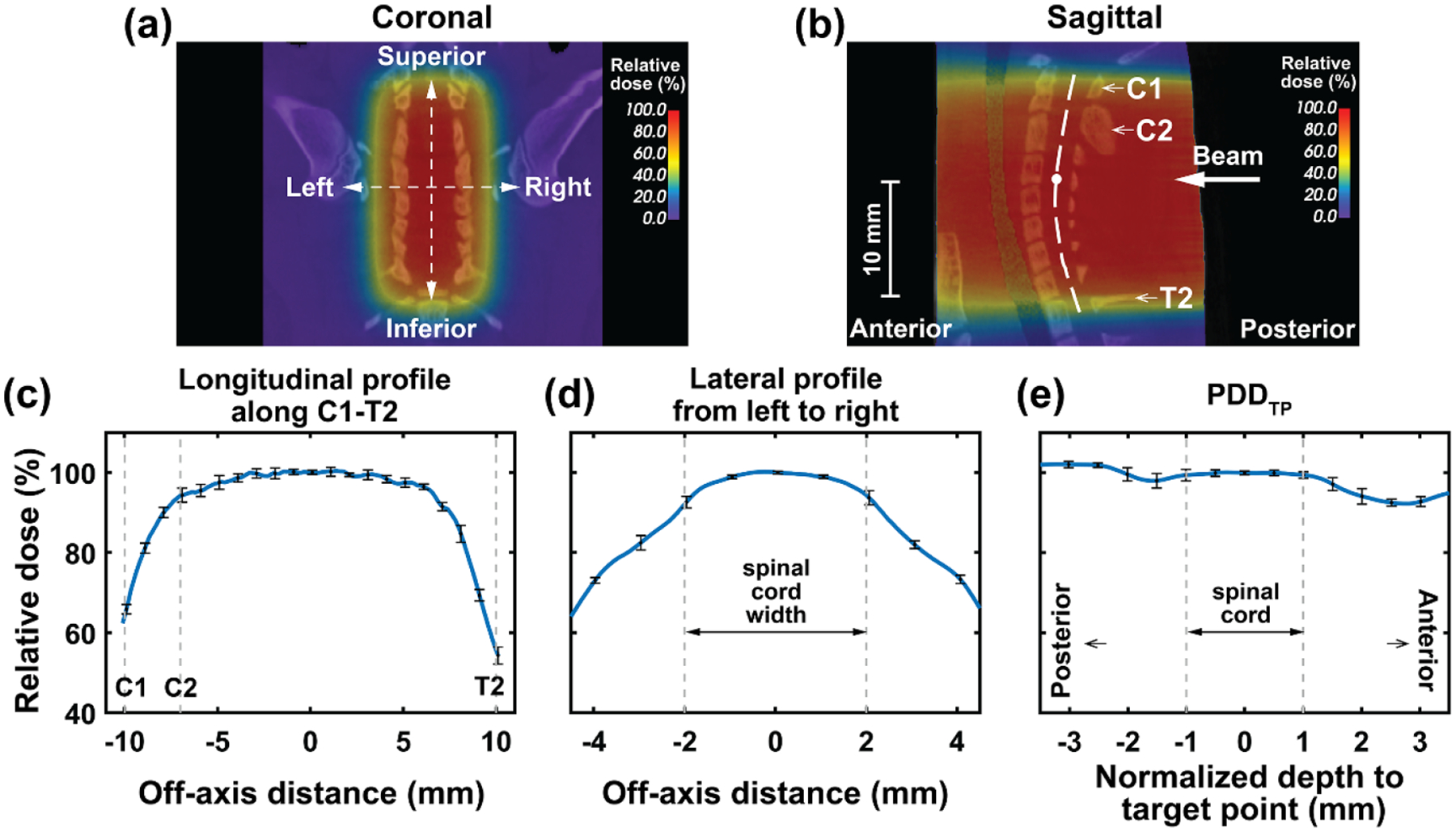
MC dosimetry of a single PA 18 MeV UHDR beam delivered to the rat C1–T2 spinal cord using a 2 × 1 cm^2^ field. (_a) and (b) show the dose distribution for a representative rat in coronal and sagittal views, respectively. The white dot marks_ the target point, located 10 mm cranial to T2 vertebra. Dose distributions are normalized to the dose at the target point. (c) Longitudinal profile along the C1–T2 spinal cord, corresponding to the white dashed curve in panel (b). (d) Lateral profile in the left-right direction. (e) PDD_TP_ with the target point defined at 0 mm. Data in panels (c)–(e) are the average of the 8 non-irradiated rats, with error bars indicating inter-animal standard deviations.

The MC-calculated dose distribution for a representative non-irradiated rat is shown in figures 4(a) and (b). Figures 4(c)–(e) present the averaged longitudinal and lateral profiles, and PDD_TP_ from the 8 non-irradiated rats, with standard deviation <3%. This low deviation justifies the use of cohort-averaged dose distributions to represent the dose distribution of individual rats of the same age and similar weight, and confirms the reproducibility of our experimental setup.

On average, the 18 MeV UHDR beam achieved uniform irradiation across a 13 mm segment of the C1–T2 spinal cord, with <5% dose variation (figures 4(b) and (c), ±6.5 mm from the 0 point in figure 4(c)). This avoids the dose-volume effect (Bijl *et al* 2002) and supports reliable assessment of the potential FLASH sparing effect. In the lateral direction, the averaged profile maintained >91% within the ~4 mm width of the spinal cord (figure 4(d)). The averaged PDD_TP_ remained >96% within the ~2 mm across spinal cord along the posterior and anterior axis (figure 4(e)), indicating uniform dose penetration. These MC results demonstrate that shoot-through delivery using an 18 MeV beam with a 2 × 1 cm^2^ field provides consistent and uniform dose coverage of the C1–T2 spinal cord in rats of the same age and similar weight.

#### 3.3.3. Comparison of UHDR and CONV dose distributions

**Figure 5.**
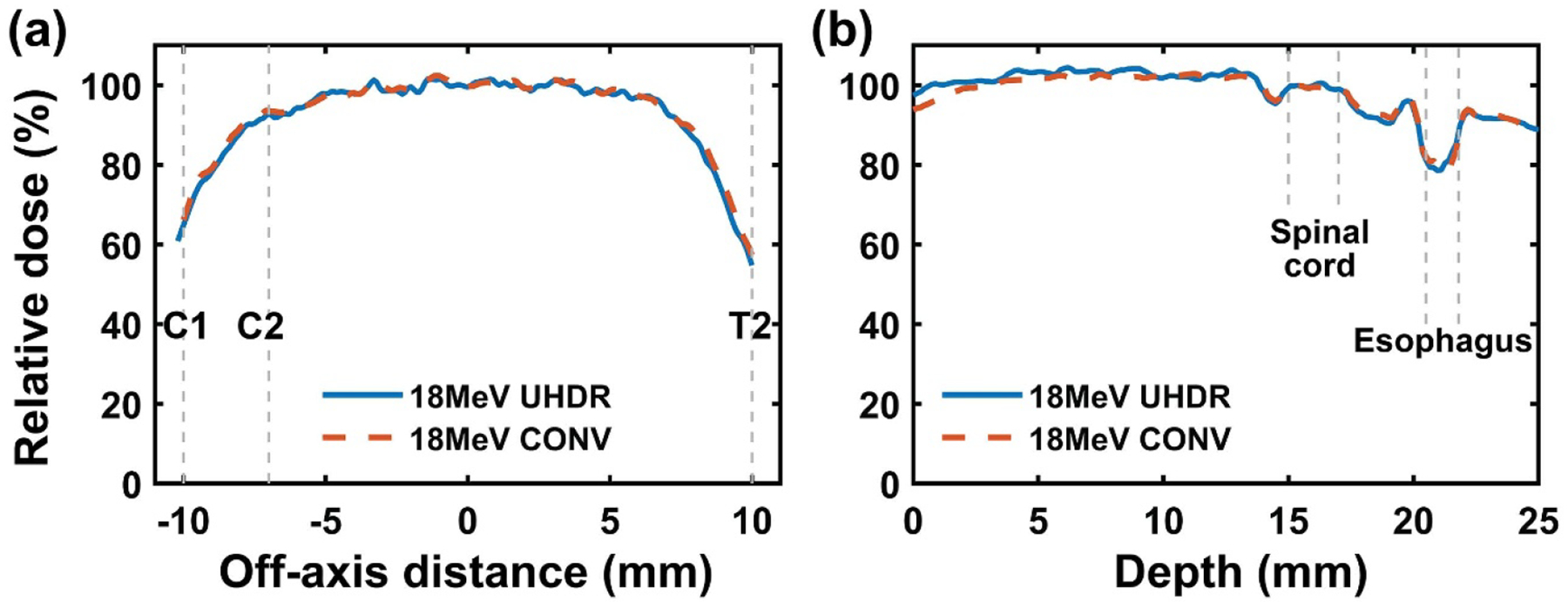
Comparison of MC dosimetry for a representative rat between the UHDR and CONV irradiation. (a) Dose profile along the C1–T2 spinal cord. (b) PDD along CAX. The posterior surface of the rat is defined at 0 mm depth.

To comprehensively evaluate the effects of FLASH-RT compared to CONV-RT, MC dose distributions were also calculated for CONV irradiation and compared to those of UHDR. Figures 5(a) and (b) present the longitudinal dose profile along the C1–T2 spinal cord and the PDD along the CAX, respectively, for both beams in a representative animal. The two beams exhibited highly similar profiles and PDDs with average absolute differences below 3%. This similarity is likely attributable to their closely matched energy-related beam characteristics, together with the reduced influence of lateral profile differences under the small field size or cutout used in this study (supplementary table S1 and figures S4, S5). Both UHDR and CONV beams achieved uniform irradiation across the ~13 mm C1–T2 segment (<5% non-uniformity) and maintained >98% of the target dose throughout the spinal cord depth range (15–17 mm from rat back surface, figure 5(b)). These findings confirm that comparable dose delivery can be achieved under UHDR and CONV conditions, thereby supporting the validity of the experimental design in isolating dose rate as the primary variable for evaluating potential dose rate-dependent biological effects.

In the shoot-through irradiation geometry, the esophagus is the primary organ at risk (OAR) within the beam path (figure 4(b)), receiving approximately 80% of the target dose (figure 5(b)). This suggests the potential need to monitor radiation-induced esophagitis during the follow-up period of the subsequent irradiation study.

### 3.4. *In vivo* MC dose and dose rate calculation validated by scintillator dosimetry

**Figure 6.**
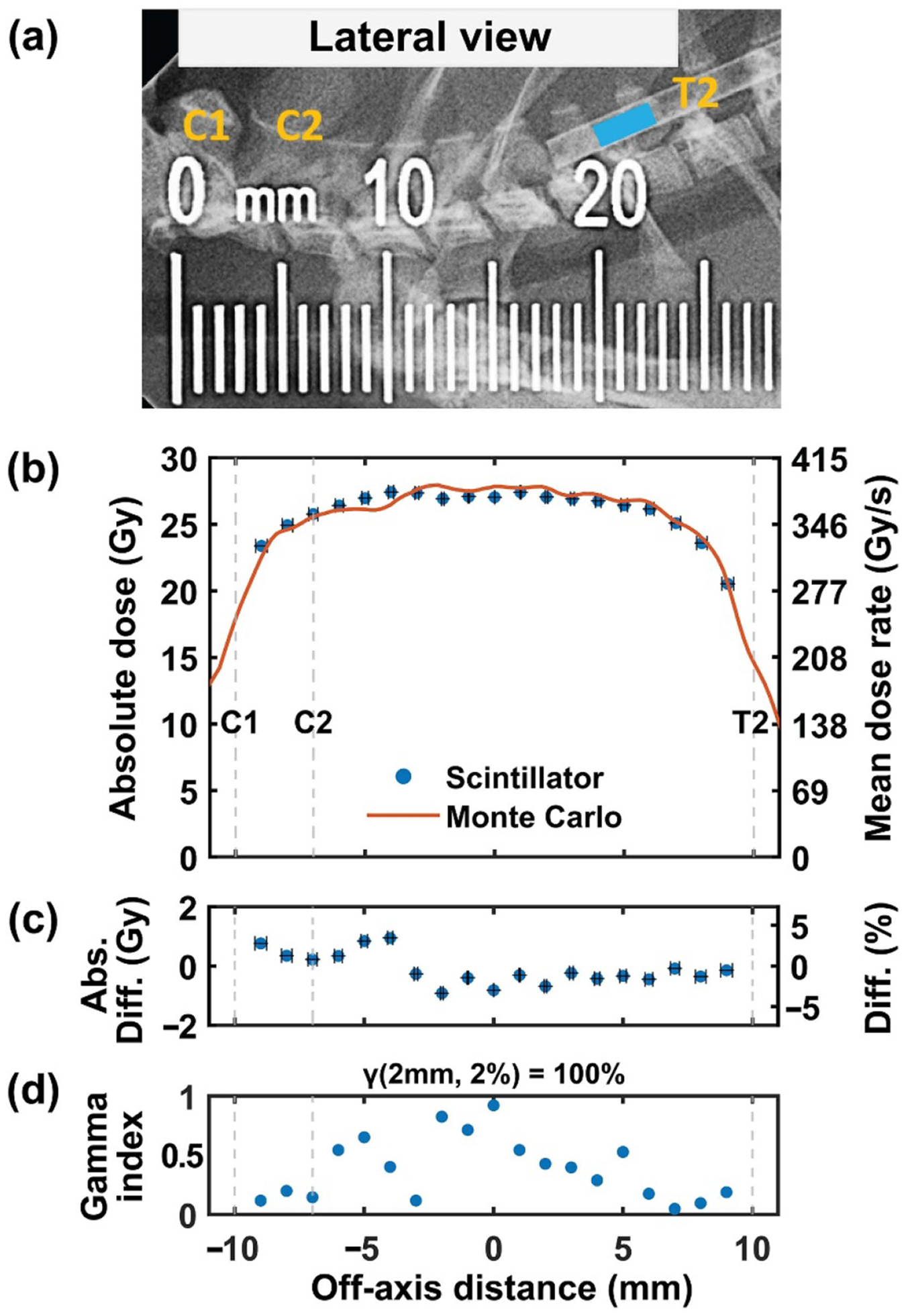
(a) X-ray imaging of the scintillator inserted into the C1–T2 spinal cord via a 6 Fr catheter. The blue square indicates the detector volume. (b) Comparison between scintillator measurements and MC calculations for the dose and dose rate along the C1–T2 spinal cord. The horizontal error bars of the scintillator measurements represent a 2.5% uncertainty in probe positioning, as the linear stage measures the scintillator’s curved path along the spinal cord, while the OAD position is projected. The scintillator measurements are within standard deviation of 0.07 Gy. (c) Corresponding absolute and percentage difference in dose between measurements and MC calculations. (d) Corresponding gamma indices as a function of OAD using 2 mm/2% criteria, with a 100% passing rate.

The MC-calculated dose and dose rate along the C1–T2 spinal cord were further verified with the scintillator measurements (figures 6(a) and (b)). The differences were <4% within the ±10 mm off-axis distance (OAD, figure 6(c)). Considering an estimated 2.5% uncertainty in probe positioning, which arises because the linear stage measures the scintillator’s curved trajectory along the spinal cord while the OAD position is projected (supplementary figure S6), the gamma passing rate was evaluated and reached 100% under the 2 mm/2% criteria (figure 6(d)). These results confirm the high accuracy of the MC dose engine and the precise dose delivery of the FLASH system.

## 4. Discussion

The goals of this project are to optimize a research platform dedicated to FLASH rat spinal cord studies and to use this platform to determine whether FLASH-RT can mitigate the spinal cord toxicity compared to CONV-RT, thereby establishing its potential to mitigate late-responding tissue toxicity and assess its clinical applicability. Although the rat spinal cord has long served as a classical model for radiation-induced toxicity (Ang *et al* 1984, Scalliet *et al* 1989, Ruifrok *et al* 1994, Bijl *et al* 2002, Philippens *et al* 2004, Karger *et al* 2006), our study introduces several key advancements going beyond traditional approaches and addressing unique challenges of UHDR *in vivo* irradiation. These include the image-guided spinal cord localization, real-time dose monitoring during UHDR delivery, MC dose assessment for both *in vivo* UHDR and CONV irradiation, and scintillator validation of MC dose and dose rate in the rat spinal cord setup.

Specifically, we first ensured accurate C1–T2 setup and localization by using the 3D-printed rat immobilization device in combination with the portable x-ray imaging (figures 1(a)–(d)). The 2D imaging-based localization was validated against CBCT imaging, showing submillimeter accuracy (table 1). Intra-fractional motion was assessed by comparing T2 positions on pre- and post-irradiation x-ray images, again showing submillimeter deviation. In the S–I direction, uncertainties of this magnitude are unlikely to affect biological outcomes for the 20 mm long C1–T2 irradiation, as consistent ED_50_ values have been reported across different spinal levels as long as the irradiated length exceeds 8 mm to avoid the dose-volume effect (Hopewell *et al* 1987, Ruifrok *et al* 1994, Bijl *et al* 2002, Philippens *et al* 2004). In the L–R direction, the observed 0.2 ± 0.1 mm offset, arising from image-guided localization uncertainty or intra-fractional motion, corresponds to <0.2% dose variation from the OAD = 0.position (figure 4(d)), preserving uniform spinal cord dose coverage. It should be noted that the immobilization device was aligned using the LINAC light-field crosshair. Alignment based on the crosshair is considered accurate within 1 mm of the beam center. When this uncertainty is combined with the image-guided localization accuracy (table 1) using root-sum-square error propagation, the overall irradiation accuracy is estimated to be <1.2 mm, which is not expected to meaningfully influence the dose-response relationship as ED_50_ values are consistent across different spinal levels when the irradiated length exceeds 8 mm. Together, our image-guided spinal cord setup and IC-based real-time dose monitoring (figure 1(e)) ensure accurate and reproducible C1–T2 localization and precise UHDR dose delivery.

To demonstrate the hypothesized superiority of FLASH-RT in sparing normal tissue toxicity, studies must be conducted under controlled conditions, using comparable UHDR and CONV plans and deliveries, with dose rate as the only variable. While many prior studies on spinal cord toxicity under CONV-RT have emphasized biological endpoints (van der Kogel 1979, Ang *et al* 1984, Scalliet *et al* 1989, Wong *et al* 1992, Ruifrok *et al* 1994, Debus *et al* 2003, Philippens *et al* 2004, Karger *et al* 2006), few have characterized the physical dose distribution in detail (Bijl *et al* 2002). Moreover, it has been recommended to report detailed dose/dose rate distributions to ensure reproducibility in FLASH research (Böhlen *et al* 2024). This motivated us to develop an MC dose engine to evaluate 18 MeV UHDR and CONV beams. Considering this, we have developed a Geant4-GAMOS MC dose engine to provide accurate dose calculations for electron beams where we explicitly modeled the head geometry of the LINAC. For the electron FLASH mode, the target and the scattering foil were removed from the LINAC geometry. The MC-calculated PDD and profiles agreed with measurements with average absolute difference <2% (figure 3), confirming the accuracy of the MC beam model and supporting its use for spinal cord dosimetry.

Our MC simulations further demonstrate that the 18 MeV UHDR beam achieved uniform irradiation (<5% non-uniformity) along the central ~13 mm spinal cord segment (figure 4(c)), avoiding the known dose-volume effect (Bijl *et al* 2002). Meanwhile it maintained >91% and >96% of the target dose across the spinal cord width (figure 4(d)) and along the PA direction (figure 4(e)), respectively. These results demonstrate the uniform dose coverage of using high-energy 18 MeV electrons for the rat spinal cord study. It should be noted that the spinal cord dose-response relationship for cervicothoracic segment with CONV-RT has been extensively studied using photons, protons, and high-energy electrons in a shoot-through beam arrangement. The corresponding ED_50_ is consistently ~21 Gy for single-fraction irradiation across all modalities (Ang *et al* 1984, Hopewell *et al* 1987, Scalliet *et al* 1989, Ruifrok *et al* 1994, Bijl *et al* 2002). This suggests that spinal cord toxicity is independent of beam modality, thereby confirming the validity of our study design of employing 18 MeV electron beams. Furthermore, our CONV beam exhibited dose distribution close to that of the UHDR beam, with differences <3% (figure 5), supporting our study design in isolating the dose rate as the only variable for evaluating FLASH-dependent biological effects.

When using a rat cohort of the same age and similar weight, inter-animal variability in dose profiles and PDD_TP_ is minimal (<3%, figures 4(c)–(e)). This approach greatly simplifies the workflow and *in vivo* dose calculation, as the averaged dose distribution can represent that of individual irradiated rats, eliminating the need for CBCT-based MC calculations for each subject. To further validate the precision of our FLASH platform, we innovatively employed a small scintillator with a high 1000 Hz sampling frequency (Guo *et al* 2025) to verify MC-calculated dose and dose rate distribution in real-time during UHDR delivery within the spinal cord, representing the first such application in our field (figure 6). The differences between the scintillator-measured dose and MC calculations were <4%, and the gamma passing rate was 100% under the 2%/2 mm criteria. These results confirm the accuracy of the MC dose engine and demonstrate that our platform can achieve the desired dose rates and dose distributions for high-precision spinal cord studies under both UHDR and CONV conditions.

Beyond dosimetric accuracy and delivery precision, experimental throughput is a key practical consideration for the applicability of a FLASH platform in large-cohort radiobiological studies. For an experienced operator, the total time required to complete the standard irradiation setup and UHDR delivery for a single rat is approximately 15–20 min, corresponding to a typical throughput of ~24 rats per experimental day (e.g. 3 dose levels with 8 rats per dose level). The image-guided positioning and repositioning phase represents the primary source of time variability and typically accounts for approximately 5–10 min of the total procedure time. Post-irradiation recovery generally requires an additional 3–5 min; however, preparation and anesthesia induction of the next animal can be performed concurrently to improve overall throughput. While not designed as a high-throughput system, this level of efficiency provides a practical and scalable throughput suitable for cohort-based FLASH radiobiological studies.

With this shoot-through delivery (figures 4(b) and 5(b)), the esophagus is the primary OAR and receives ~80% of the target dose. Although transient esophagitis with early weight loss (⩽2 weeks) could occur, prior studies have shown rapid recovery thereafter (van der Kogel 1979, Wong *et al* 1992, Debus *et al* 2003), suggesting limited impact on study outcomes. We will monitor weight change post-irradiation of our study cohort and provide supportive care as needed. Our upcoming biological outcomes will be reported separately.

## 5. Conclusion

We developed the first comprehensive, image-guided preclinical platform capable of operating in both UHDR and CONV modes to investigate FLASH-RT-mitigated spinal cord toxicity using a rat model. The system integrates pulse control, real-time output monitoring, custom immobilization, precise image-guided target localization, and *in vivo* MC dosimetry validated against scintillator measurements. Using this platform, we can achieve accurate and reproducible dose delivery at both UHDR and CONV dose rates for rat spinal cord irradiation. This work thus establishes a robust foundation for systematic evaluation of the FLASH effect in late-responding organs and for determining clinical applicability of FLASH-RT.

## Supplementary Material

supplementary material

Supplementary material for this article is available online

## Figures and Tables

**Table 1.** Image-guided localization accuracy of the 2D x-ray based setup for rat spinal cord irradiation was evaluated by CBCT, while intra-fractional motion was assessed using pre- and post-irradiation x-ray images.

	Direction	Mean ± standard deviation (mm)
2D x-ray localization accuracy relative to CBCT.	S–I	0.3 ± 0.3
	L–R	0.2 ± 0.1
Intra-fractional motion between pre- and post-irradiation x-ray image	S–I	0.3 ± 0.6
	L–R	0.2 ± 0.1

## Data Availability

All data that support the findings of this study are included within the article (and any supplementary information files). Supplementary materials available at https://doi.org/10.1088/1361-6560/ae56cb/data1.
