# Supplementary material for: A high-precision image-guided platform for studying spinal cord toxicity under ultra-high dose rate electron irradiation
Source: Phys Med Biol. Author manuscript; Available in PMC 2026 Jun 9. (PMC13249001; doi:10.1088/1361-6560/ae56cb)
Supplement: supplementary material [file NIHMS2181490-supplement-supplementary_material.pdf]

Supplementary material

Illustration of T2 vertebra localization on CBCT and 2D X-ray images

Figure S1 illustrates the localization of T2 vertebra relative to the central radiopaque ruler using 3D CBCT and 2D X-ray imaging. Along the superior–inferior (S–I) direction, the T2 position on the ruler was read directly from the 2D X-ray image (e.g., 33.2 mm in Fig. S1b) or determined from the coronal and sagittal views of CBCT images using 3D Slicer (e.g., 33.4 mm in Fig. S1c and d). Along the left–right (L–R) direction, the lateral distance between the center of the T2 vertebra, represented by the yellow line in the CBCT coronal view (Fig. S1c) or the center of the orange circle in the 2D X-ray image (Fig. S1b), and the distal end of the radiopaque ruler representing the midline of the immobilization device (red dots in Fig. S1b and c), was measured using 3D Slicer for CBCT images (e.g., 0.2 mm in Fig. S1c) or the built-in imaging software of the X-ray detector for 2D X-ray images (e.g., 0.3 mm in Fig. S1b).

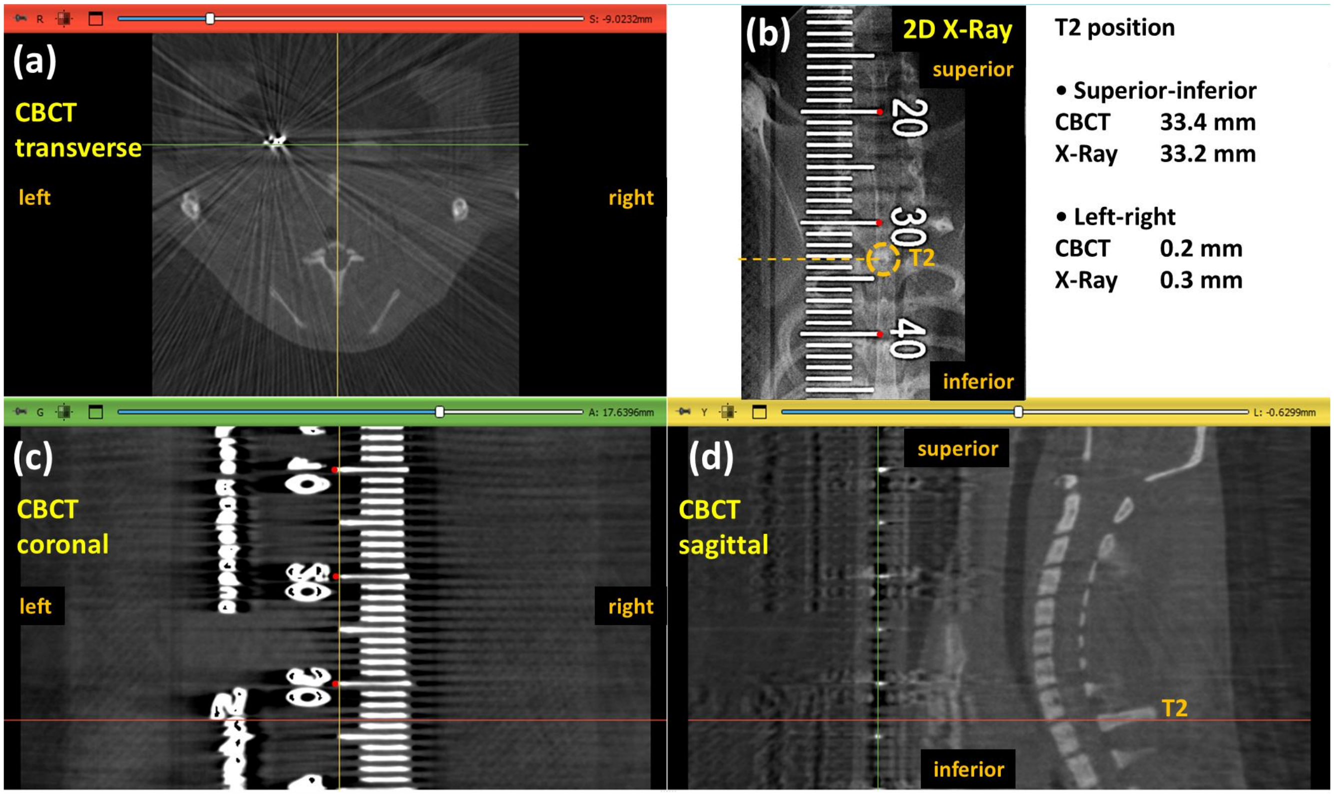

Figure S1. Illustration of T2 vertebra localization relative to the central radiopaque ruler using 3D CBCT (a, c, and d) and 2D X-ray imaging (b).

Illustration of ion chamber positioning for UHDR output monitoring

Figure S2 shows that the ion chamber was secured to the distal end of the electron cone using tape for UHDR output monitoring. The ion chamber position was kept fixed for all experimental sessions.

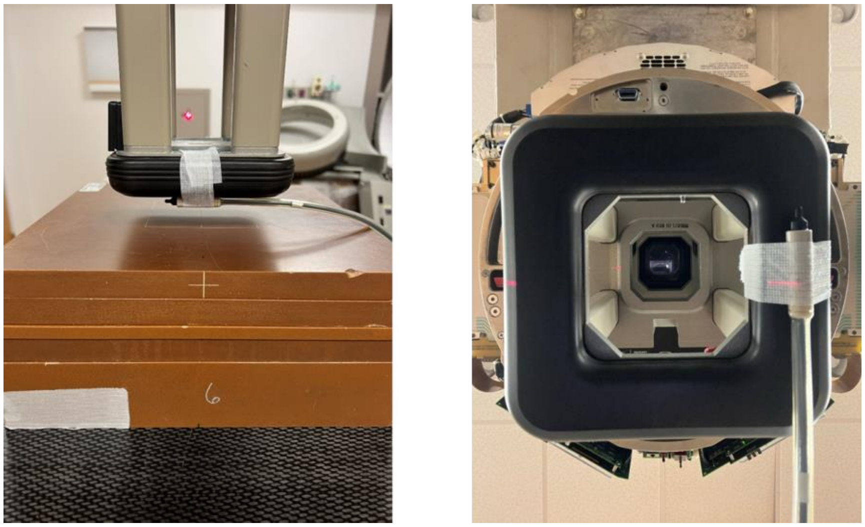

Figure S2. Positioning of the ion chamber on the distal end of the electron cone to measure Bremsstrahlung and scattered radiation in real-time, serving as a surrogate for UHDR output monitoring.

Illustration of raw scintillator signal resolving individual pulses of 18 MeV UHDR beam

Figure S3 shows raw scintillator signals capable of resolving individual pulses of an 18 MeV UHDR beam delivered at a pulse repetition frequency of 180 Hz.

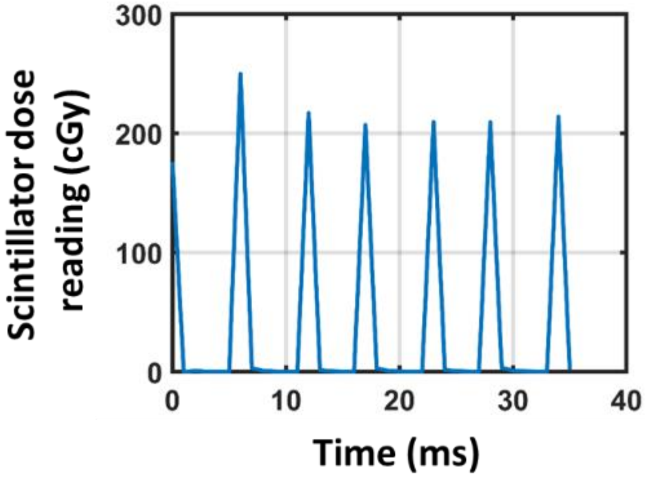

Figure S3. Raw scintillator signal showing pulse-to-pulse temporal structure of a 7-pulse 18 MeV FLASH electron beam delivered at a pulse repetition frequency of 180 Hz.

Explanation of the close agreement between 18 MeV CONV and UHDR in vivo dose distributions

To our knowledge, the close agreement in the in vivo dose distributions between the CONV and FLASH beams (Fig. 5 in the manuscript) can be primarily attributed to two factors: (1) the similarity in their energy-related beam characteristics, and (2) the minimized impact of lateral beam profile differences under the small field sizes used in this study.

As shown in Table S1, the energy-related beam characteristics of the 18 MeV CONV and FLASH beams under the 10 × 10 cm^2 cone are closely matched. Consistent with these results, the corresponding PDDs (Fig. S4) show good agreement between the two beams, except in the bremsstrahlung region, where the FLASH beam exhibits reduced photon contamination due to the absence of a scattering foil.

In-air beam profiles were further compared under an open field setting at 95 cm SSD (Fig. S5). While the CONV 18 MeV electron beam exhibits a flattened profile and the FLASH electron beam converted from 18 MV photon mode follows a Gaussian distribution in the absence of flattening filter, the profile difference within the small field size used in this study (2 × 1 cm^2) is minimized to less than 5%. As a result, the lateral dose distributions of the two beams are highly similar within the central beam region, with the 2 × 1 cm^2 cutout in place, contributing to the close agreement observed in the in vivo dose distributions (Fig. 5a in the manuscript).

Table S1. Beam characteristics of the 18 MeV CONV and FLASH beams under the 10×10 cm^2 cone.

Field size:<br>10×10 cm^2 cone	18 MeV CONV	18 MeV FLASH
d_{max} (cm)	2.6	2.5
R_{80} (cm)	6.2	6.2
R_{50} (cm)	7.6	7.4
R_p (cm)	9.2	9.1
$(E_p)_0$ (MeV)	18.6	18.4

Abbreviations: (1) d_{max}: Depth of maximum dose; (2) R_{80}: Depth at which the dose falls to 80% of the maximum dose; (3) R_{50}: Depth at which the dose falls to 50% of the maximum dose; (4) R_p: Practical range of electrons; (5) $(E_p)_0$: Mean energy of the electron beam at the phantom surface.

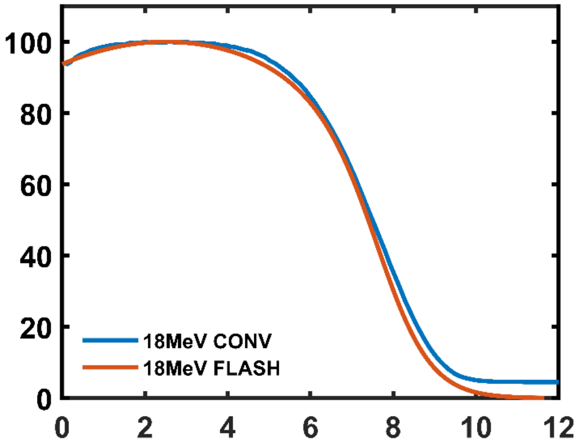

Figure S4. PDDs of the 18 MeV CONV and FLASH beams under the 10×10 cm^2 cone.

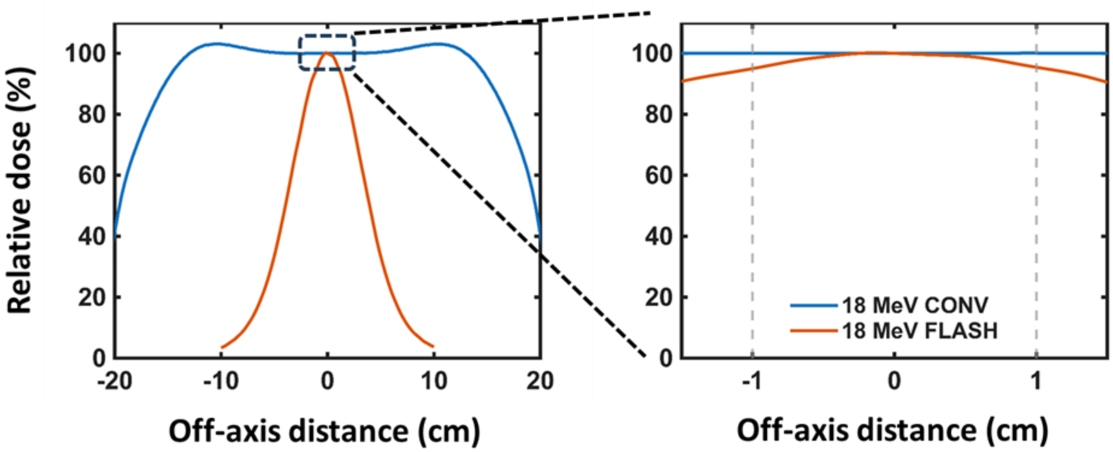

Figure S5. Crossline in-air profiles of the 18 MeV CONV and FLASH beams under the open field setting.

Illustration of scintillator movement path along the spinal canal

Figure S6 illustrates the actual curved movement path of the scintillator within the spinal canal of a rat carcass compared with the projected off-axis distance (OAD) used for profile analysis in Fig. 6 of the manuscript.

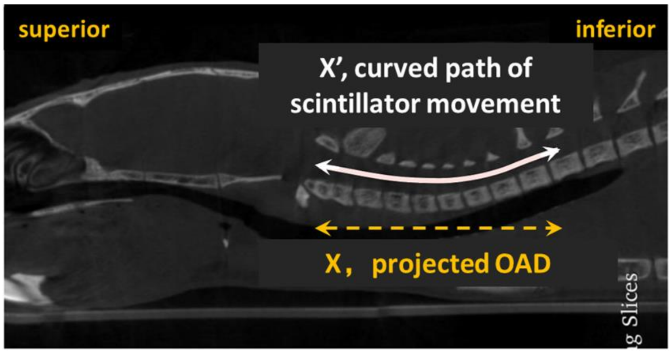

Figure S6. CT image of a rat illustrating the curved path of scintillator movement (X') compared to the projected OAD (X) used for profiles. The ratio X'/X was determined close to 1.025.
